# The oxygen reserve index (ORI): a new tool to monitor oxygen therapy

**DOI:** 10.1007/s10877-017-0049-4

**Published:** 2017-08-08

**Authors:** T. W. L. Scheeren, F. J. Belda, A. Perel

**Affiliations:** 1 Department of Anaesthesiology, University of Groningen, University Medical Center Groningen, PO Box 30 001, 9700 RB Groningen, The Netherlands; 2 Department of Anesthesiology, Hospital Clínico Universitario, Valencia, Spain; 3 Department of Anesthesiology and Intensive Care, Sheba Medical Center, Tel Aviv University, Tel Aviv, Israel

**Keywords:** Monitoring, Oxygenation, Pulse oximetry, Hypoxia, Hyperoxia, Oxygen therapy, Critical care, Perioperative period, Operating rooms, Preoxygenation, Hypoxemia, Hyperoxemia, Hyperoxic acute lung injury

## Abstract

Supplemental oxygen is administered in the vast majority of patients in the perioperative setting and in the intensive care unit to prevent the potentially deleterious effects of hypoxia. On the other hand, the administration of high concentrations of oxygen may induce hyperoxia that may also be associated with significant complications. Oxygen therapy should therefore be precisely titrated and accurately monitored. Although pulse oximetry has become an indispensable monitoring technology to detect hypoxemia, its value in assessing the oxygenation status beyond the range of maximal arterial oxygen saturation (SpO_2_ ≥97%) is very limited. In this hyperoxic range, we need to rely on blood gas analysis, which is intermittent, invasive and sometimes delayed. The oxygen reserve index (ORI) is a new continuous non-invasive variable that is provided by the new generation of pulse oximeters that use multi-wavelength pulse co-oximetry. The ORI is a dimensionless index that reflects oxygenation in the moderate hyperoxic range (PaO_2_ 100–200 mmHg). The ORI may provide an early alarm when oxygenation deteriorates well before any changes in SpO_2_ occur, may reflect the response to oxygen administration (e.g., pre-oxygenation), and may facilitate oxygen titration and prevent unintended hyperoxia. In this review we describe this new variable, summarize available data and preliminary experience, and discuss its potential clinical utilities in the perioperative and intensive care settings.

## Background

Oxygen administration can be life-saving since lack of oxygen (hypoxia) is deleterious and should be prevented or treated in a timely manner. This is why the vast majority of patients in the operating room and in the intensive care unit (ICU) receive supplementary oxygen. However, oxygen has to be considered as a drug since the administration of too much oxygen and the presence of hyperoxia are known to be associated with significant complications [1]. Oxygen therapy should therefore be precisely titrated and accurately monitored to avoid the consequences of both hypoxia and hyperoxia [2]. Pulse oximetry has become the prevalent monitoring technology to detect the presence of hypoxemia [3]. However, in order to monitor the oxygenation status when the arterial blood is nearly fully saturated (SpO_2_ ≥ 97%) we need to rely on blood gas analysis, which is intermittent and invasive and occasionally delayed. New technological developments in multiwavelength pulse co-oximetry expand our abilities to monitor oxygenation in the moderate hyperoxic range (PaO_2_ 100–200 mmHg). In this review we will describe this new variable, termed the Oxygenation Reserve Index (ORI), and discuss its potential clinical utilities.

## The problem of hypox(em)ia

Although low oxygen tensions (PaO_2_ values <75 mmHg or <10 kPa) are frequently observed (e.g. at high altitude) and usually well tolerated by healthy individuals, they are deleterious for patients who undergo major surgery or during critical illness. It should also be noted that oxygen tension is considerably lower at the capillary or mitochondrial level than it is in the atmosphere, alveoli or arterial blood (the “oxygen cascade”) [4].

In the perioperative setting, both hypoxia (i.e. lack of oxygen at the tissue level) and hypoxemia (i.e. low blood oxygen level) occur commonly and may be considered a serious safety concern. For instance, a recent report analysing 95,407 anaesthesia records from 2 institutions revealed that 6.8% of patients had a hypoxemic event (defined by oxygen saturation, SpO_2_ <90%) during the intraoperative period, and that 3.5% of patients had a severely hypoxemic event (SpO_2_ <85%) lasting 2 min or longer [5]. The risk factors for the occurrence of such events include obesity [6, 7], difficult airway [8], rapid sequence induction [9], unrecognized esophageal intubation [8], one-lung ventilation [10], and very young age [7, 11]. Hypoxemic events have been found to be even more frequent in the early postoperative period in the post anaesthesia care unit (PACU). In a prospective blinded observational study including 1500 postoperative patients, 37% had at least one period of hypoxemia (SpO_2_ <90%) lasting ≥1 h, while in 11% of patients such hypoxemia lasted ≥6 h [12]. Furthermore, 8% of patients spent ≥5 min/h with an SpO_2_ <85%, and 3% spent ≥30 min with an SpO_2_ <80% [12]. Another retrospective analysis of 137,757 patients in the PACU revealed that many of the hypoxemic events occurred ≥30 min after PACU admission, i.e. when the anaesthesiologist has already left the patient and returned back to the OR for the next case [13]. Importantly, about 25% of these hypoxemic episodes lasted >10 min after their onset [13]. The first postsurgical day was similarly found to carry the highest risk of adverse respiratory events, most of them being hypoxic episodes [14]. Risk factors for postoperative hypoxemia include residual effects of anaesthetics, opioids or muscle relaxants [15], atelectasis [16], upper abdominal or thoracic surgery [17], smoking, pulmonary diseases, obesity and advanced age [18–20].

In general, a clear association between progressively lower PaO_2_ and increasing in-hospital mortality has been demonstrated also in ICU patients [21]. For instance, airway problems may occur in the ICU setting as well [22], and lead to particularly adverse outcome [23]. A recent study on 97,844 mechanically ventilated patients across 165 ICUs in the US revealed that mortality was increased in patients extubated overnight compared to those being extubated at daytime [24].

## The problem of hyperox(em)ia

Exposure to pure O_2_ for longer periods provokes pulmonary inflammation (hyperoxic acute lung injury, HALI, or “O_2_-toxicity”), but in the absence of high-stretch mechanical ventilation, the risk of HALI is minimal when the FiO_2_ is ≤0.6, and the risk probably begins when the FiO_2_ exceeds 0.7 for several hours (≥24 h). However, resorption atelectasis, which increases right-to-left blood shunting and deteriorate pulmonary gas exchange, may appear within minutes after induction of anaesthesia and increase postoperative pulmonary complications [25]. Likewise, a high FiO_2_ might cause respiratory depression in situations where respiratory drive becomes oxygen dependent, such as in patients with COPD or in postoperative patients with atelectasis and residual pain medication.

Furthermore, hyperoxia (high PaO_2_) decreases cardiac output by decreasing heart rate and causing systemic vasoconstriction [26]. Furthermore, hyperoxia is a potent vasoconstrictor stimulus to the coronary circulation, functioning at the level of the microvascular resistance vessels. This is why the hyperoxia related to routine use of high-flow oxygen in uncomplicated myocardial infarction may result in a greater infarct size and possibly increase the risk of mortality [27]. Yet the use of supplemental oxygen is widespread in cardiac patients, exposing them to significant periods of potential detrimental hyperoxia without monitoring oxygen tension [28]. It is noteworthy in this context that a lower FiO_2_ had no worse outcomes during cardiac surgery when compared to the higher concentrations that are commonly used in this setting [29].

Finally, it has recently been shown in volunteers that normobaric hyperoxia decreased capillary perfusion and muscle oxygen consumption and increased microcirculatory perfusion heterogeneity [30]. Since such microcirculatory alterations are independently associated with worse patient outcomes [31], the use of hyperoxia as a therapeutic strategy may be deleterious. Last but not least a very recent study has shown in 480 ventilated ICU patients that a conservative oxygen treatment causing only small differences in PaO_2_ (87 vs. 102 mmHg) might cause a significant mortality benefit (11 vs. 20%) [32].

Despite these negative effects, unintended hyperoxia seems to be quite common in daily ICU practice. According to a large Dutch study including 5498 mechanically ventilated patients, hyperoxia (PaO_2_ >120 mmHg) was found in 22% of the arterial blood gas samples, but the FiO_2_ was subsequently decreased in only 25% of these instances, implying that hyperoxia was accepted [33]. In fact, another study found that ICU patients spent most of their time (59%) in hyperoxemia, and when this occurred at an FiO_2_ between 0.3 and 0.4, no FiO_2_ adjustments were made in 88% of these episodes [34]. Liberal oxygenation is also common in the pediatric ICU [35], despite existing guidelines to the contrary.

When looking at the relation between oxygen therapy and outcome, a recent study involving 14,441 ICU patients showed that severe hyperoxia (PaO_2_ >200 mmHg) was associated with higher mortality rates (odds ratio 1.35) and fewer ventilator-free days compared to both mild hyperoxia (PaO_2_ 120–200 mmHg) and normoxia. However, the adjusted probability of in-hospital death showed its lowest values at PaO_2_ values between 120 and 160 mmHg [36]. Similar results were obtained in a previous study observing more than 36,000 patients from 50 ICUs in the Netherlands that revealed a U-shaped relationship between mortality and arterial PO_2_, with a nadir at PaO_2_ values of 15–20 kPa (110–150 mmHg) [37]. Similar results have been reported recently in two major trials including more than 5000 and 6000 patients after resuscitation from cardiac arrest, respectively: mortality sharply increased both at PaO_2_ values <9 kPa (<67 mmHg) and >30 kPa (>225 mmHg) [38], and hyperoxia was independently associated with increased in-hospital mortality [39]. In addition, a recent meta-analysis of 16 studies covering more than 49,000 patients revealed a crude odds ratio of 1.38 (95% CI 1.18–1.63) (*p* < 0.0001) for in-hospital mortality when patients were hyperoxic, independent of admission diagnosis [40]. However, a review of 152,680 ventilated ICU patients demonstrated an association between hypoxia but not hyperoxia and increased in-hospital mortality during the first 24 h [21].

So according to the evidence it is quite clear that hyperoxemia in critically ill patients is not beneficial and may increase mortality and that the optimal PaO_2_ is not yet clearly defined. However, there may be situations where short periods of hyperoxia may actually increase patient safety, e.g. during the induction of anaesthesia and for intubation in ICU patients to delay the onset of hypoxemia during apnea [41, 42]. In fact, various maneuvers have been proposed to extend the effect of preoxygenation, including the use of high-flow nasal oxygen [43–45].

Finally, there is some experimental evidence on the potential benefit of hyperoxia during resuscitation from hemorrhagic shock [46] but not in septic shock [47].

## Monitoring oxygenation by pulse oximetry

**Fig. 1:**
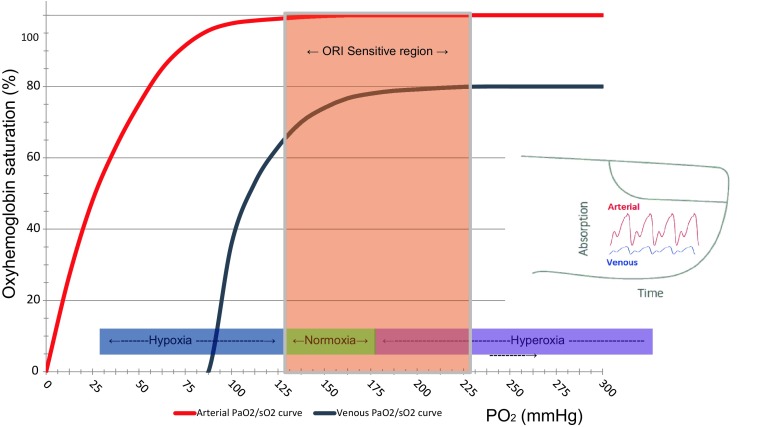
Arterial (*red line*) and venous (*blue line*) oxyhaemoglobin dissociation curves. In the hypoxic rage (PaO_2_ <100 mmHg), arterial oxygenation can be assessed by pulse oximetry (SpO_2_). As PaO_2_ increases beyond 100 mmHg, venous saturation (SvO_2_) at the measurement site increases even though arterial saturation (SaO_2_) remains maximal and unchanged. This change in SvO_2_ causes changes in absorption of the incident light (and hence a change in measured signals) as PaO_2_ changes. With Masimo’s Rainbow SET technology these signals are extractable and the system is able to detect changes in PaO_2_ through changes in SvO_2_ at the measurement site. SvO_2_ reaches a plateau beyond a certain level of PaO_2_, approximately 200 mmHg (hyperoxic range), and consequently ORI is sensitive to the changes in PaO_2_ in the range between 100 and 200 mmHg (*orange shaded area*)

Arterial blood gas analysis (aBGA) is considered the gold standard of monitoring the oxygenation status. It does, however, require arterial blood sampling, which is intermittent, invasive, associated with blood loss, costly, and the results are often delayed. As a result, significant changes in oxygenation may go unnoticed in-between aBGA’s when the arterial oxygen saturation (SaO_2_) is close to 100% and therefore no longer informative (Fig. 1).

The most common method to monitor oxygenation is the non-invasive continuous pulse oximetry which has become a universal standard of care in anaesthesia, intensive care medicine, and other patient populations who are susceptible to become hypoxemic [3]. Pulse oximetry measures hemoglobin oxygen saturation (SpO_2_) by light absorption typically at the finger or the earlobe. The accuracy of pulse oximetry as compared with invasive measurements is good with a bias between SpO_2_ and SaO_2_ <2% and a precision <3% [3].

It is of interest to note that although the routine monitoring of SpO_2_ with pulse oximetry has become mandatory in anaesthesia and critical care, there is no formal evidence-base showing its positive impact on patient outcome [48].

Nevertheless, and in agreement with current strong conviction, the relative number of respiratory complications during anaesthesia and associated malpractice claims has dropped significantly and consistently since the introduction of pulse oximeters [49, 50].

Pulse oximetry has a major limitation when it comes to assessing or preventing hyperoxemia in patients that receive oxygen therapy. Due to the sigmoid shape of the oxyhemoglobin dissociation curve, SpO_2_ is near complete at a PaO_2_ of 90–100 mmHg, and increasing the PaO_2_ above this level will no longer affect the SpO_2_. As a consequence, when SpO_2_ is ≥97%, the PaO_2_ level could be anywhere between 90 and 600 mmHg. Hence, only a direct measurement of the PaO_2_, as part of aBGA, can be used to assess the hyperoxic range. Another way to assess oxygenation status in the hyperoxic range is by gradually decreasing the inspired oxygen concentration (FiO_2_) until SpO_2_ starts to decrease, as recently suggested [51]. This maneuver, however, takes several minutes and may culminate in undesirable low SpO_2_ levels, making the procedure impractical in cases where the clinical situation does not allow to decrease the FiO_2_. As a result of these limitations, the monitoring of SpO_2_ alone cannot identify clinically important changes that may occur in the oxygenation status in the hyperoxic range, nor can it rule out the presence of unintended hyperoxia in patients receiving oxygen therapy.

Finally, pulse oximetry alone is a late detector of respiratory depression even when associated with considerable CO_2_ retention if supplemental oxygen is being administered [52].

## New development: the oxygen reserve index (ORI)

The oxygen reserve index (ORI) is a new development in multiple wavelength pulse oximetry that reflects, in real-time and non-invasively, the oxygenation status in the moderate hyperoxic range (PaO_2_ of approximately 100–200 mmHg, see shaded area in Fig. 1) in patients receiving supplemental oxygen. The measurement of this new variable has been made possible by the ability of multiwave length pulse co-oximetry (Rainbow SET, Masimo Inc., Irvine, Ca., USA) to analyse both arterial and venous pulsatile blood absorption changes of incident light in a finger. When oxygen is being administered, the PaO_2_ increases to >100 mmHg and the SpO_2_ maximizes at close to 100%. However, the venous oxygen saturation (SvO_2_) at the measurement site continues to increase until it stabilizes (at about 80% saturation) when the PaO_2_ reaches about 200 mmHg (Fig. 1). By combining the Fick and oxygen content equations, the resulting change in light absorption over this PaO_2_ range is the basis for the ORI calculation. Hence, the ORI is an index with a unit-less scale between 0.00 and 1.00, which is a relative indicator of changes in PaO_2_ in the moderate hyperoxic range (approximately 100–200 mmHg), and is aimed for use in patients receiving supplemental oxygen [53]. It has to be stressed that ORI is not equivalent to PaO_2_ and cannot replace arterial blood gas analysis and/or ‘classic’ pulse oximetry. For further information about the principles of measurement and calculation of the ORI the interested reader is referred to reference [53] and the appendix of reference [54].

## Preliminary results from the perioperative setting

There are two clinical studies published as of yet demonstrating that the ORI may provide an early warning when arterial oxygenation deteriorates before any changes in SpO_2_ occur. In the first study, Szmuk et al. followed the ORI during induction of anaesthesia in 25 healthy children [54]. They found that ORI detected an impeding desaturation in median of 31.5 s (range 19–34.3 s) before changes in SpO_2_ occurred and concluded that this represents a clinically important warning time, which might give clinicians time for corrective actions. This statement has been emphasized by the accompanying editorial, acknowledging a promising role of ORI for patient safety [55]. The second study looked at the relationship between ORI and PaO_2_ in 106 patients undergoing surgery and found a significant positive relationship for both absolute values (*r* ^2^ = 0.536) and changes of both variables (*r* ^2^ = 0.421) in the PaO_2_ range up to 240 mmHg [56].

**Fig. 2:**
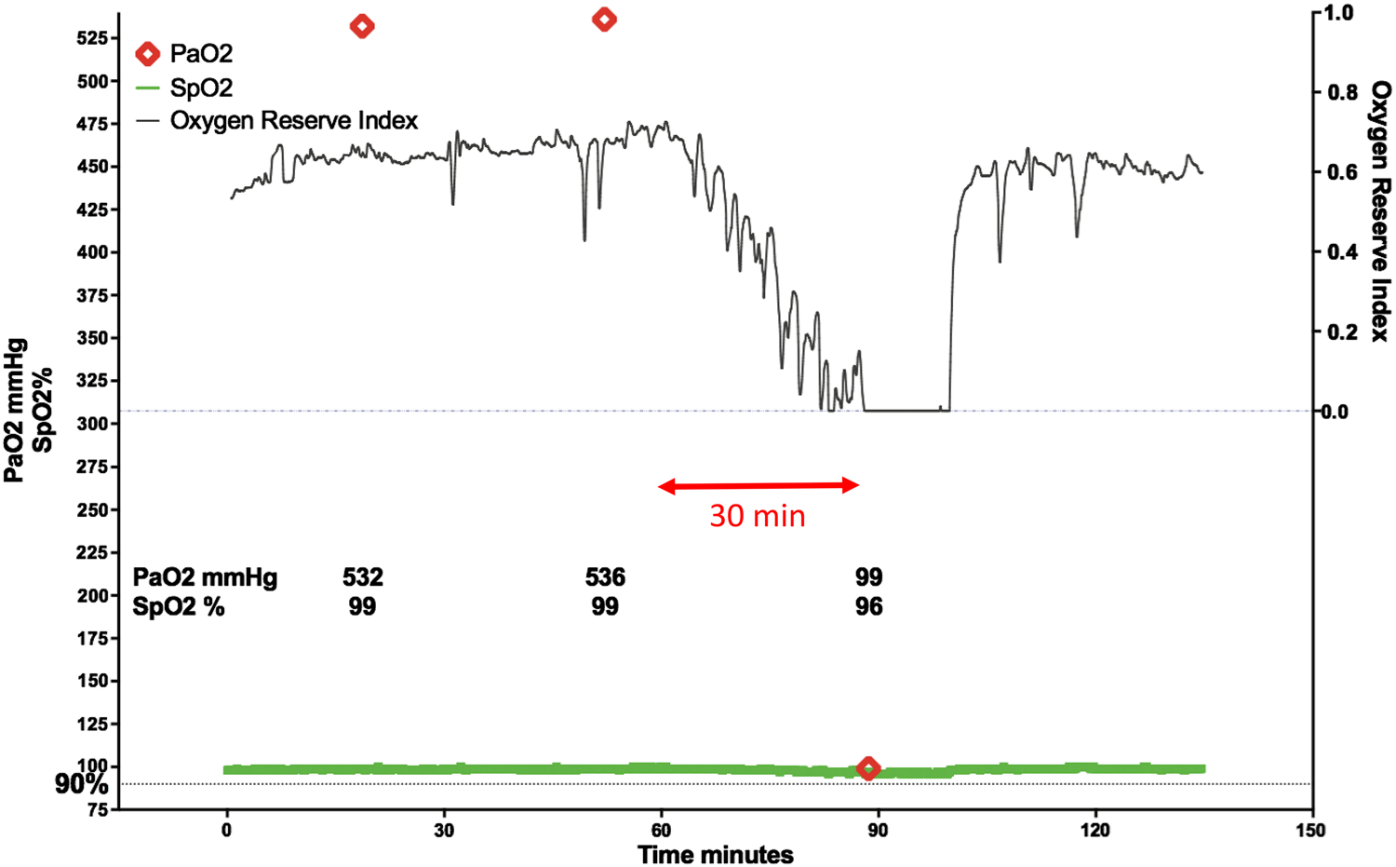
Example of continuous intraoperative oxygen reserve index trend (ORI; *black line*), continuous pulse oxygen saturation trend (SpO_2_; *green line*), and intermittent arterial partial pressure of oxygen determination (PaO_2_; *red diamonds*) obtained during surgery. ORI decreased during 30 min before aBGA documented a large decrease in PaO_2_. Reprinted with permission from [56]

As illustrated in a plot obtained from an individual patient undergoing surgery (Fig. 2), the ORI (black line) indicated a decrease in oxygenation 30 min before the next aBGA (red diamond) revealed that the PaO_2_ had in the meantime decreased from 500 to 100 mmHg while the SpO_2_ (green line) remained unchanged [56].

**Fig. 3:**
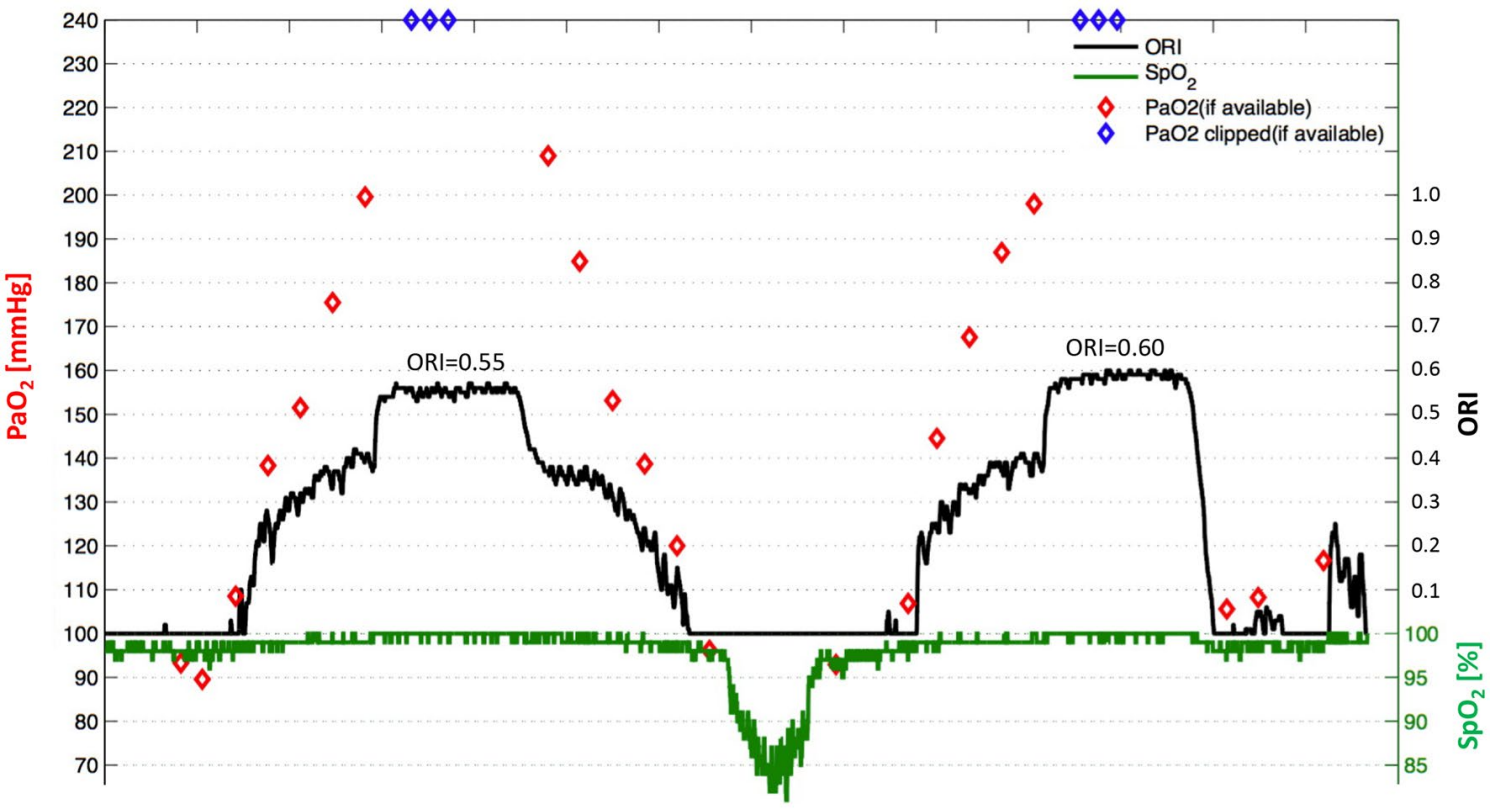
Representative example from a volunteer breathing via a tight-fitting facemask standardized oxygen concentrations ranging from normoxia (FiO_2_ 0.21) to hyperoxia (FiO_2_ 1.0) and mild hypoxia (FiO_2_ 0.14) in a stepwise fashion

Recently, we performed a validation study of ORI in healthy volunteers, preliminary results of which have been presented at the Euroanaesthesia 2016 meeting in London [57]. Volunteers were breathing via a tight fitting facemask standardized oxygen concentrations in a stepwise fashion, ranging from ambient air (FiO_2_ 0.21) to pure oxygen (FiO_2_ 1.0), followed by a short period of mild hypoxia (FiO_2_ 0.14). As shown in the representative example (Fig. 3), the ORI followed closely the changes in PaO_2_ related to changes in FiO_2_, peaking at values of 0.55 and 0.60 respectively with maximum FiO_2_. A later gradual decrease in FiO_2_ led to a decrease in ORI values, which reached zero just before the SpO_2_ (green line) started to decrease following the breathing of the hypoxic FiO_2_. Our results clearly show that the ORI reflects changes in oxygenation due to changes in FiO_2_, and that a decline in ORI following a decrease in FiO_2_ occurs well before any changes in SpO_2_ occur. It is important to note that in a recently revised version of the disposable pulse oximeter sensor (code M rainbow R1), the ORI has been rescaled so that it will reach the maximum value of 1.0 in more patients.

## The potential clinical utility of the ORI

### Response to oxygen administration and to preoxygenation

Since the ORI has been introduced into clinical practice only recently, we will provide a theoretical exploration of its possible clinical utility in patients who receive supplemental oxygen. First of all, the ORI may provide information regarding the patient’s initial response to acute oxygen therapy [58]. This information may be of great importance, since the persistence of a low ORI in spite of a high FiO_2_ may reflect the presence of high intrapulmonary shunting, ventilation/perfusion mismatch or hypoventilation. The most common example of V/Q mismatch is atelectasis related to induction of anaesthesia. A major cause of anaesthesia-induced lung collapse in this period is the use of high inspired oxygen concentration. Loss of muscle tone and muscle paralysis, compression of lung tissue due to heart weight and loss of surfactant function reduces functional residual capacity, producing gas trapping and resorption atelectasis behind closed airways. All these mechanisms are well reviewed elsewhere [59–61].

**Fig. 4:**
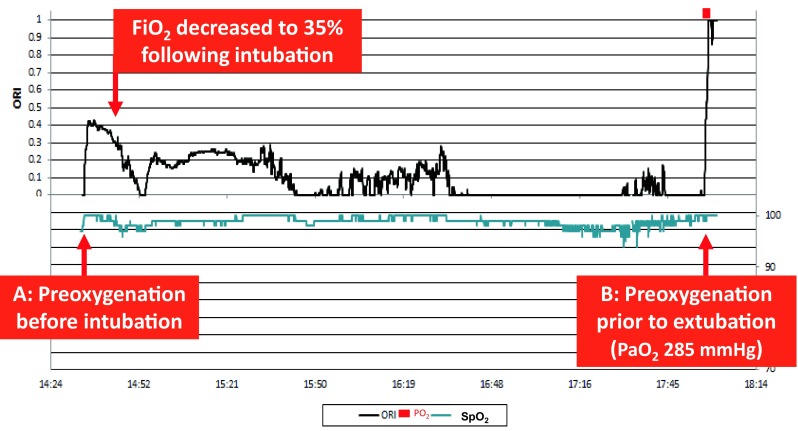
Clinical graphic example of the ORI response in a patient receiving supplemental oxygen before tracheal intubation (pre-oxygenation) (*A*) and again prior to extubation (*B*). In both instances, the ORI sharply increases, making the effects of pre-oxygenation visible. Following intubation, the FiO_2_ is reduced and titrated so that the ORI remained slightly above zero, indicating sufficient oxygenation of the arterial blood and avoiding hyperoxia at the same time. With kind permission from Drammen Sykehus, Norway

The ORI may also prove useful during the pre-oxygenation that is routinely done before endotracheal intubation and extubation and whenever there is an anticipated interruption in oxygen delivery (Fig. 4). The importance of performing preoxygenation correctly has been recently described again [42, 44, 62].

By increasing pulmonary oxygen reserve, well-performed preoxygenation may extend the ‘tolerable apnea time’, defined as the time until the SpO_2_ reaches 90%, to almost 10 min [62], and hence make endotracheal intubation safer. In patients with normal pulmonary function, a well-performed preoxygenation can be easily tracked by the response of the ORI (Fig. 4). However, in obese patients and in the critically ill, preoxygenation by breathing 100% O_2_ alone may not achieve the expected increase in oxygen reserve, and further interventions like positive-pressure ventilation and CPAP may be necessary [62, 63]. The ORI may therefore be useful in identifying those patients who do not increase their oxygenation in response to preoxygenation, as reliance on the SpO_2_ only may be misleading in such cases [63]. Monitoring of ORI may also help in identifying a faulty preoxygenation technique, including a low or absent fresh oxygen flow, presence of gas-leaks due to an ill-fitting mask, shallow breathing, too short pre-oxygenation period, etc.

### Better titration of FiO_2_

The ORI may be a useful tool for the correct titration of the FiO_2_. Although in the critically ill, ventilated patients pulse oximetry may be useful in maintaining patients above the hypoxemic levels by adjusting inspired oxygen fractions and ventilator settings [51], the SpO_2_ per se may not exclude the presence of unintended hyperoxia without further time-consuming manipulations of the FiO_2_ or measuring aBGA. As stated before, unintended hyperoxia occurs very frequently in both adult and pediatric ICU patients [33–35].

### Early warning of impending hypoxemia

The recent Prospective Evaluation of a RIsk Score for Postoperative Pulmonary COmPlications in Europe (PERISCOPE) study showed that about 4.2% of surgical patients develop postoperative respiratory failure, carrying an in-hospital mortality of 10.3% (vs. 0.4% in the rest of the patients), and defined risk factors for development of postoperative respiratory failure [64]. Such susceptible high-risk surgical patients, including those who undergo bariatric surgery [65], may benefit from earlier detection of postoperative respiratory failure. These patients often receive oxygen therapy which, as said before, renders routine pulse oximetry to be an inadequate tool for an early detection of developing hypoxemia and/or hypoventilation, so that postoperative monitoring with ORI should be considered.

The ORI might also be used to monitor oxygenation during high-flow nasal cannula oxygenation, used in patients who are not or no longer intubated and mechanically ventilated, in obese patients or during bronchoscopy [66]. Particularly the increasing number of obese patients admitted to the ICU postoperatively or requiring long-term non-invasive ventilation (NIV) might be a future indication for ORI monitoring in order to prevent respiratory failure [67] or unintended hyperoxia.

Several recently published trials would have benefitted from the use of ORI if it had been available, such as two RCTs of apneic oxygenation during endotracheal intubation of the critically ill [68, 69], the CLOSE study comparing conservative versus a liberal oxygenation targets for mechanical ventilation [70], or the OPERA trial looking at the effects of high-flow nasal cannula oxygen therapy to prevent hyperoxia after major abdominal surgery [71].

### Immediate response to changes in PEEP and to recruitment manoeuvres

**Fig. 5:**
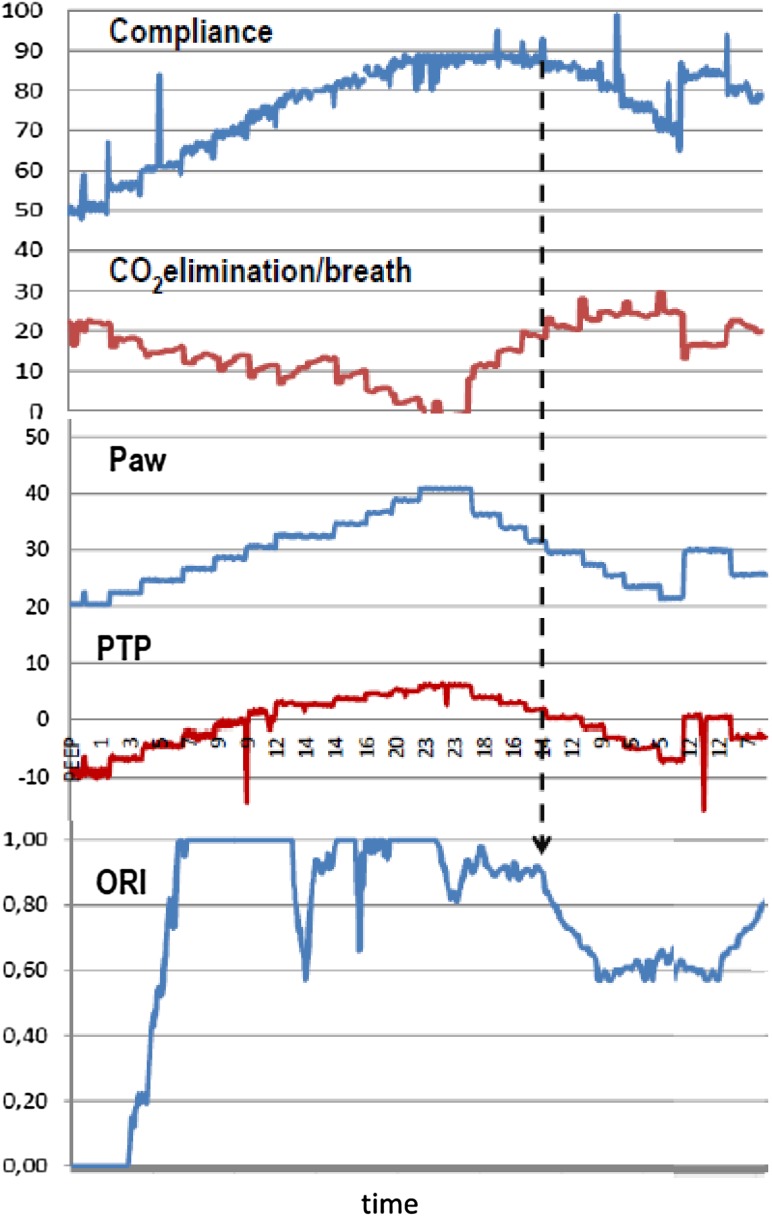
Representative example of simultaneous trends in Compliance (ml/cmH_2_O), CO_2_ elimination per breath (ml), airway pressure (Paw, cmH_2_O), transpulmonary pressure (PTP, cmH_2_O) and ORI values of a patient during a recruiting maneuver (RM). While Paw rises in steps at the beginning of the RM, PTP and compliance rise accordingly and ORI increases up to a maximum value. Increases of dead space due to distention reduces the CO_2_ elimination per breath. During the decremental PEEP trial, step-reductions in Paw and PTP do not reduce compliance or ORI values until a closing point is observed. The *dotted arrow* shows this point in which an abrupt drop in compliance and ORI coincide with a negative value of TPT indicating re-collapse of the lung. The optimal PEEP corresponds to the previous setting

The ORI may reflect the immediate response of the PaO_2_ to therapeutic measures like adjusting positive end-expiratory pressure (PEEP) and recruitment manoeuvres. In a recent pilot study we have examined the effects of recruiting maneuvers on the ORI in ICU patients [72]. As can be seen in Fig. 5, the incremental increase in airway pressure during the maneuver is closely followed by an increase in the ORI. During the decremental PEEP trial (for finding the optimal PEEP) the ORI maintains a high value until the closing point appears where a sharp decrease in ORI is observed. This example demonstrates that the ORI may be a useful non-invasive tool for following oxygenation at any FiO_2_ during recruiting maneuvers and determining the optimal PEEP for an individualized lung-protective strategy in ICU patients.

### Possible limitations of the ORI

Although the ORI may reflect oxygenation in the moderate hyperoxic range, it should not be considered as equivalent to PaO_2_ and is certainly not meant to replace pulse oximetry (SpO_2_). The ORI should be considered as complementing SpO_2_ for monitoring the oxygen status in patients receiving supplemental oxygen. Since the ORI measurement and calculation are affected by a variety of factors (e.g., pH, temp, PaCO_2_, etc.) it has a significant inter-individual variability. In addition, most individuals will not reach an ORI above 0.6–0.7 (at least with the first version of the ORI sensor) when breathing at an FiO_2_ close to 1.0, so that an individualized scaling of the maximum ORI being reached while breathing pure oxygen might be useful. Finally, like pulse oximetry (SpO_2_), the ORI may not read when peripheral perfusion (at the finger) is impaired, such as in shock states or during high-dose vasopressor therapy.

### ORI summary

**Fig. 6:**
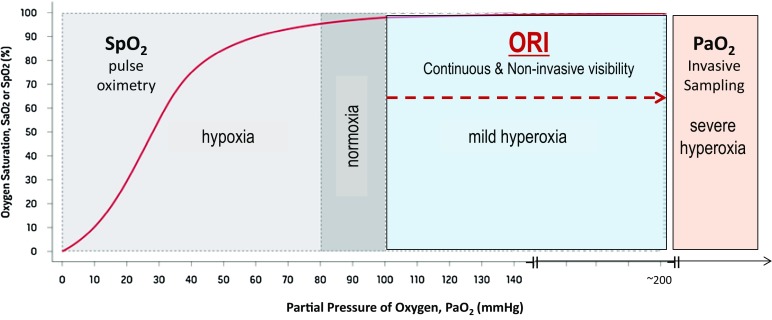
Oxyhaemoglobin dissociation curve showing the potential usefulness of the continuous and non-invasive oxygen reserve index (ORI). The ORI has its place in the mild hyperoxic range (PaO_2_ 100–200 mmHg). For lower values, pulse oximetry for monitoring SaO_2_ is useful, for higher values, invasive sampling and arterial blood gas analysis for measuring PaO_2_ must be used

Figure 6 summarizes the place that the ORI could take in monitoring the patient`s oxygenation status, namely the mild hyperoxic rage (PaO_2_ 100–200 mmHg). Below this range, pulse oximetry can easily be used to detect a decrease in SpO_2_. For higher PaO_2_ values, invasive arterial blood sampling and aBGA must be used.

## Conclusions

The ORI is a new promising variable that serves as a relative indicator of the PaO_2_ in the range of 100–200 mmHg. Its addition to conventional pulse oximetry opens new opportunities in the continuous, non-invasive monitoring of the oxygenation status in patients receiving supplemental oxygen. The ORI may potentially allow better control of pre-oxygenation, provide an alarm of decreasing oxygenation well before any decrease in SpO_2_, allow a more adequate titration of oxygen therapy and prevent unintended hyperoxia, and reflect the immediate impact of recruitment maneuvers and PEEP adjustments. Further studies are needed to determine the potential role of the ORI in the management of acutely ill patients who are in need of oxygen supplementation.
