# Correction to: The oxygen reserve index (ORI): a new tool to monitor oxygen therapy

**DOI:** 10.1007/s10877-018-0104-9

**Published:** 2018-02-14

**Authors:** T. W. L. Scheeren, F. J. Belda, A. Perel

**Affiliations:** 1 Department of Anaesthesiology, University of Groningen, University Medical Center Groningen, PO Box 30 001, 9700 RB Groningen, The Netherlands; 2 Department of Anesthesiology, Hospital Clínico Universitario, Valencia, Spain; 3 Department of Anesthesiology and Intensive Care, Sheba Medical Center, Tel Aviv University, Tel Aviv, Israel

## Correction to: J Clin Monit Comput 10.1007/s10877-017-0049-4

In the original publication of the article, the authors have realized an error in Fig. 1. The corrected version of Fig. 1 is given below.

**Fig. 1:**
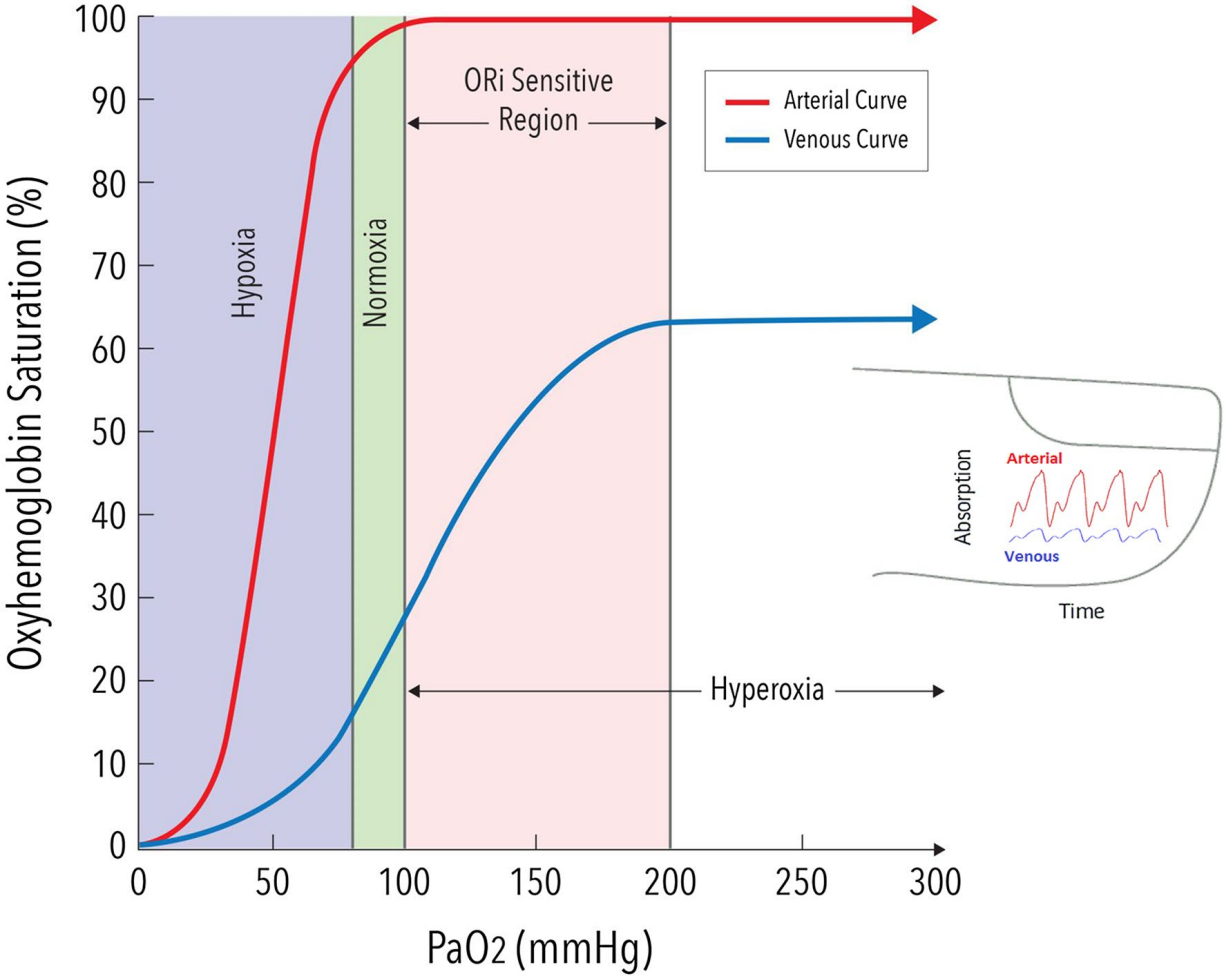
Schematic representation of arterial (red line) and venous (blue line) oxyhaemoglobin dissociation curves. In the hypoxic rage (PaO_2_ < 100 mmHg), arterial oxygenation can be assessed by pulse oximetry (SpO_2_). As PaO_2_ increases beyond 100 mmHg, venous saturation (SvO_2_) at the measurement site increases even though arterial saturation (SaO_2_) remains maximal and unchanged. This change in SvO_2_ causes changes in absorption of the incident light (and hence a change in measured signals) as PaO_2_ changes. With Masimo’s Rainbow SET technology these signals are extractable and the system is able to detect changes in PaO_2_ through changes in SvO_2_ at the measurement site. SvO_2_ reaches a plateau beyond a certain level of PaO_2_, approximately 200 mmHg (hyperoxic range), and consequently ORI is sensitive to the changes in PaO_2_ in the range between 100 and 200 mmHg (orange shaded area)

